# Potentially preventable incidence of diabetes due to risk factor modification

**DOI:** 10.1186/2251-6581-11-8

**Published:** 2012-08-24

**Authors:** Seyed Mohammad Kalantar Motamedi, Reza Majdzadeh, Fatemeh Ardeshir Larijani, Fakher Raheem, Zahra Koleini, Bagher Larijani

**Affiliations:** 1 Endocrinology and Metabolism Research Center, Tehran University of Medical Sciences, P.O.Box: 1411413137, Tehran, Iran; 2 Knowledge Utilization Research Center, School of Public Health, Tehran University of Medical Sciences, Tehran, Iran; 3 School of Medicine, Tehran University of medical sciences, Tehran, Iran

**Keywords:** Diabetes mellitus, Potential impact fraction, Prevention

## Abstract

**Background:**

Increasing diabetes incidence demands investigation of risk factors, prioritization and designing modification interventions. We calculated the potential modifiable incidence of diabetes due to reduction in risk factors.

**Methods:**

We used counterfactual analysis model to estimate avoidable burden of incident diabetes related to each risk factor. The potential impact fraction (PIF) index calculated utilizing the data of current prevalence, magnitude of impact and counterfactual status of risk factors. We considered the levels of evidence while giving higher priority to domestic data.

**Results:**

The estimated PIF regarding minimum feasible risk for the impaired fasting glucose (IFG), impaired glucose tolerance (IGT), combined IFG/IGT, low HDL, high triglyceride, high total cholesterol, hypertension, general obesity, central obesity and physical inactivity were 0.13, 0.10, 0.18, 0.01, 0.12, 0.03, 0.13, 0.03, 0.02 and 0.10, respectively.

**Conclusion:**

While the combined risk factors of IFG and IGT should be noticed as the most important potential factor in prevention of diabetes and reducing its incidence burden, among the other risk factors, modification of hypertension, high triglyceride, and physical inactivity could have more impact.

## Introduction

Diabetes is considered as one of the top 10 leading causes of death in middle-income countries, it has reached to seventh place in the high-income countries [1]. According to the International Diabetes Foundation 285 million people are diabetics worldwide, that will rise to 438 million by 2030 [1].This increase in the diabetes incidence in African and Middle-East countries are more than the industrialized countries [1]. In Iran the burden of diabetes was calculated about 306440 years in year 2000 [2]. It has been estimated that the prevalence of diabetes will rise from 8% to 9.8%, from 2010 to 2030 [1]. This fact represents an increased effect of costs of diabetes in the future. Therefore, due to numerous and costly complications of diabetes, designing suitable interventions to control this process would be necessary. To design more effective or efficient interventions and prioritize them, the preventive health systems programs, should calculate an indicator called potential impact fraction (PIF) of risk factors. The counterfactual analysis model was introduced as a suitable estimation of modification for incident disease prior to preventive interventions [3]. This index predicts the rate of change in incidence of disease following a change in any of the risk factors.

Counterfactual analysis model, which has shown its efficiency in the analysis of disease risk factors in global burden study, is a suitable basis for prioritization of preventive health systems programs. In this model, the current situation of the risk factor (prevalence) is called factual, the target condition of that factor (target prevalence for the health system) is called counterfactual [4-8], and avoidable burden of the disease which is obtained via primary preventive programs (reducing the prevalence of risk factor) is called PIF [3,4,7-12]. In order to calculate the incidence of PIF, the factual, strength of the impact and counterfactual are required.

The counterfactual can fall into one of the four conditions including theoretical minimum risk level, minimum level of reasonable risk, feasible minimum risk levels or minimum levels of cost-effectiveness. We, however, selected only the feasible minimum risk levels.

This review aimed to determine the PIF of the risk factors in preventing and reducing the diabetes burden.

## Methods

### Definition of variables

The risk factors for diabetes included in this study were impaired fasting glucose (IFG: 100 ≤ FPG <126 mg / dl) [13], high triglycerides (TG > 250 mg / dl) [14], impaired glucose tolerance test (IGT: 140 ≤ 2hPG <200 mg / dl), low HDL (HDL <35 mg / dl for male subjects and HDL <45 mg / dl for females) [14], high total cholesterol (> 200 mg / dl T-Chol), abdominal obesity (WC > 80 in women and WC > 94 in men) [14] ,general obesity (BMI ≥ 30) [15], hypertension (SBP ≥ 140 or DBP ≥ 90) [16], physical activity (intensive exercise of less than once a week). To calculate the PIF of each risk factor, those studies were selected that used same definition and criterion of the variables of interest.

### Search strategy and data sources

We searched MEDLINE, EMBASE, SID (Scientific Information Database) and Scopus. The selected search engines searched using specific keywords such as Potential Impact Fraction (PIF), diabetes risk factors, diabetes prevention, generalized impact fraction, Iran, burden of the diabetes, dyslipidemia, hypertriglyceridemia, hypercholesterolemia, impaired glucose tolerance, impaired fasting glucose and other terms for risk factors. The general keywords included metabolic syndrome, burden of diseases, prevention, modifiable risk factors and their combinations.

### Study selection

Eligible studies were life-style modifying interventions that enrolled normal subjects of Iranian population. To determine the counterfactual prevalence of risk factors, firstly, local interventional studies that succeed to reduce the prevalence of risk factors were studied. However, in case of the absence of local studies, global literatures were used. The priority was given to those studies with the highest level of internal and external validity.

### Calculations

The data gathered from eligible studies was used to calculate the potential impact fraction for each diabetes risk factors. Prevalence of risk factors, the magnitude of their effect on increasing the risk of incidence diabetes in terms of odds ratios of relative risks and the feasible counterfactual state of the risk factor frequency was obtained and entered into the PIF formula as mentioned in the Appendix.

## Results

We used the data of Third national surveillance of risk factors of non-communicable diseases (SuRFNCD-2007) [17] to determine the prevalence of diabetes risk factors (Table 1). 4233 people aged between 25 and 64 were selected in a randomized cluster sampling from all the provinces of the country and the sample size was proportionate to total population for each province. We also extracted the odds ratios of each risk factor for diabetes. The fact that our study was a cross-sectional study; we couldn’t extract the risk of impaired glucose tolerance and impaired fasting glucose for diabetes.

**Table 1:** The prevalence of diabetes risk factors in Iran, Esteghamati et al. (17)

**Prevalence % (95% CI)**	**Variable**
16.8(16.4-17.2)	IFG(mg/dl)
22.3(20.2-24.5)	General obesity (cm)
53.6(50.4-56.7)	Central obesity (kg/m^2^)
42.9(40.4-45.4)	Hypercholesterolemia (mg/dl)
26.7(23.9-29.4)	Hypertriglyceridemia (mg/dl)
42.7(40.3-45.1)	Low HDL(mg/dl)
26.6(24.4-28.9)	Hypertension(mmhg)
40.0(37.8-42.4)	Physical inactivity

IFG: Impaired Plasma glucose. IFG: 100 ≤ FPG < 126 mg/dl.

Hypertriglyceridemia: TG > 250 mg/dl.

HDL: High- Density Lipoprotein. Low HDL in male: HDL < 35 mg/dl .Low HDL in female: HDL < 45 mg/dl.

Hypercholesterolemia: T-Chol > 200 mg/dl.

Central obesity: Female WC > 80, Male WC > 94 cm.

General Obesity: Body Mass Index (BMI) ≥30 kg/m^2^.

Hypertension: SBP ≥ 140 or DBP ≥ 90 mmHg.

Physical inactivity: ≤ 1 time exercise during the week.

In order to find the magnitude effect of the risk factors we extracted data of a 6-year follow-up of 3307 non diabetics aged above 20 years performed by Harati et al. [18]. Identification of new cases of diabetes was during two phases of the study, first between 2001 and 2005 and second between 2005 and 2008. The criteria for diabetes were adopted from American Diabetes Association definition. Definitions for risk factors with their cut-off points and metabolic syndrome, were based on ECDCDM 2003 and IDF in 2005 respectively [19,20]).

Likewise was the study of Valdes et al. [21] which is a 6-year follow-up of 1034 people of age 30 to 75. The results are summarized in Table 2.

**Table 2:** Magnitude of the effect of risk factors on the incident of diabetes

**Risk factor**	**OR Esteghamati et al.**	**OR Harati et al. (18)**	**Adjusted OR - Harati et al. (18)**	**OR - Valdez et al. (21)**	**Adjusted OR - Valdez (21)**
IFG	-	8.3 (4.2–16.5)	7.4 (3.6–15.0)	11.5 (5.6–23.6)	8.4 (3.1–23)
IGT	-	7.1 (5.1–9.8)	5.9 (4.2–8.4)	6.7 (3.4–13.3)	4.7 (1.9–11.7)
IFG + IGT	-	42.2 (25.8–75.7)	42.2 (23.8–74.9)	45.6 (15.8–131.4)	45.6 (15.8–131.4)
General Obesity	2.04(2.48-1.66)	4.0 (2.7–5.8)	2.3 (1.5–3.6)	6.1 (2–18.8)	**-**
Central Obesity	1.97(1.61-2.33)	1.4 (1.3–1.5)	1.9 (1.4–2.6)	-	**-**
Hypercholesterolemia	1.99(1.61-2.45)	-	-	1.6 (0.8–3)	**-**
Hypertriglyceridemia	2.75(2.14-3.53)	2.0 (1.5–2.6) $	1.4 (1.1–1.9)	4.8 (2.2–10.4)	**-**
Low HDL	1.19(0.97-1.46)	1.4 (1.0–1.9)	-	2.9 (1.5–5.6)	**-**
Hypertension	-	1.9 (1.4–2.6)	-	4 (2.1–7.9)	**-**
Physical Inactivity	1.5(1.24-1.82)	-	-	2.7 (1.4–5.1)	

IFG: Impaired Plasma glucose. 100 ≤ FPG <126 mg/dl.

IGT: Impaired glucose tolerance. 140 < Two-hour glucose levels ≤199 mg /dl.

Hypertriglyceridemia: TG > 250 mg/dl.

HDL: High- Density Lipoprotein. Low HDL in male: HDL < 35 mg/dl .Low HDL in female: HDL < 45 mg/dl.

Hypercholesterolemia: T-Chol > 200 mg/dl.

Central obesity: Female WC > 80, Male WC > 94 cm.

General Obesity: Body Mass Index (BMI) ≥30 kg/m^2^.

Hypertension: SBP ≥ 140 or DBP ≥ 90 mmHg.

Physical inactivity: ≤ 1 time exercise during the week.

We also searched for domestic studies in order to determine the counterfactual status of each risk factor. The only found prospective intervention for modification of risk factors was by Harati et al. in year 2010. The reduction in risk factors was only successful in diastolic blood pressure; total cholesterol and triglyceride, while others increased during follow up. Although they reported the decrease in incidence of diabetes to less than a half of that for control group, it was not the most successful intervention for modification of all the risk factors. Therefore we considered some non-domestic studies.

The Finnish diabetes Prevention Study by Lindstrom et al. [22] is a 2-year follow-up of 522 high risk subjects for diabetes. They were randomly grouped into life style intervention and control groups. Each subject in the intervention group received individualized counseling aimed at reducing weight and intake of total and saturated fat and increasing intake of fiber and physical activity. The summarized results of this study are shown in Table 3. It should be noticed that the decrease in the frequency of high risk subjects was not mentioned directly but the mean decrease in the value of each factor was reported. Considering the normal distribution of variable values in the population, we estimated the decrease of high risk people for all the factors. We did not find any study in which changes in high risk population was reported, except for hypertension. Lindstrom et al. reported the systolic and diastolic hypertension separately, so that we were unable to estimate the true number of hypertensive participants.

**Table 3:** Estimation of reduction in prevalence of high risk population in Iran, based on counterfactual minimum risk extracted from the study of Lindstrom et al

**Risk factor**	**Reduction % (95% CI)**
General Obesity	12% (10.3-13)
Central Obesity	10% (8.8-11.0)
IFG	8% (7.3-8.7)
IGT	21% (19.7- 22.1)
Hypercholesterolemia	10% (9.1- 10.6)
Hypertriglyceridemia	40% (38.2 – 43.5)
Low HDL	13% (12.1 – 14.3)
Physical Inactivity	60% (54.5 – 63.2)

IFG: Impaired Plasma glucose. 100 ≤ FPG <126 mg/dl.

IGT: Impaired glucose tolerance. 140 < Two-hour glucose levels ≤199 mg /dl.

Hypertriglyceridemia: TG > 250 mg/dl.

HDL: High- Density Lipoprotein. Low HDL in male: HDL < 35 mg/dl .Low HDL in female: HDL < 45 mg/dl.

Hypercholesterolemia: T-Chol > 200 mg/dl.

Central obesity: Female WC > 80, Male WC > 94 cm.

General Obesity: Body Mass Index (BMI) ≥30 kg/m2.

Hypertension: SBP ≥ 140 or DBP ≥ 90 mmHg.

Physical inactivity: ≤ 1 time exercise during the week.

In order to estimate the possible decrease in hypertensive people, in a 9 year follow up, Steinberg et al. [23], from 1998 through 2006 reported a decline from 4675 to 3647 cases. This is equivalent to 22% reduction in prevalence of hypertension.

We also utilized the results of the PREMIER Clinical Trial which succeeded in modifying hypertension from 38% to 12%. The subjects in this trial were not under antihypertensive therapy. Participants were randomized to one of 3 intervention groups: the "established," group was a behavioral intervention that implemented established recommendations; the “established plus DASH," which also implemented the DASH ^a^ diet; and an "advice only" comparison group.

The physical activity modification potential was estimated from a study by Laaksonen et al. [24] from Finnish Diabetes Prevention Study. They modified physical inactivity from 37% to 15%, which is about a 60% reduction in the high risk population.

Finally the potential modifiable fraction burden of the diabetes incident due to different risk factors was calculated (Table 4).

**Table 4:** Calculated “Potential Impact Fractions” of risk factors for prevention of incident diabetes

**Risk factor**	**Factual prevalence of RF**	**Estimated counter factual prevalence of RF**	**Potential impact fraction**
IFG	16.8	12.4	0.13
IGT	6.7	3.3	0.10
IFG + IGT	4.2	3.0	0.18
General Obesity	22.3	18.7	0.03
Central Obesity	53.6	50	0.02
Total Cholesterol	42.9	37.7	0.03
Triglyceride	26.7	16.2	0.12
HDL	42.7	37.1	0.01
Hypertension	26.6	8.6	0.13
Physical Inactivity	40.0	16	0.10

*RF*: Risk Factor.

*IFG*: Impaired Plasma glucose. 100 ≤ FPG <126 mg/dl.

*IGT*: Impaired glucose tolerance. 140 < Two-hour glucose levels ≤199 mg /dl.

Hypertriglyceridemia: TG > 250 mg/dl.

*HDL*: High- Density Lipoprotein. Low HDL in male: HDL < 35 mg/dl .Low HDL in female: HDL < 45 mg/dl.

Hypercholesterolemia: T-Chol > 200 mg/dl.

Central obesity: Female WC > 80, Male WC > 94 cm.

General Obesity: Body Mass Index (BMI) ≥30 kg/m2.

Hypertension: SBP ≥ 140 or DBP ≥ 90 mmHg.

Physical inactivity: ≤ 1 time exercise during the week.

## Discussion

Since the index of “Attributable Risk” seems inappropriate to estimate the possible reduction of the disease and gives impractical values, a more objective and operational index is necessary to check the conditions of the disease in case of risk factors modification. The importance of this study is that it is the first attempt to observe the modifiable burden of incident diabetes by reducing risk factors using the potential impact fraction (PIF). Moreover, risk factors cannot completely and practically be removed from the society, but to a certain extent are modifiable. Therefore, the extent of feasible, plausible or cost-effective modification minimum risk addition to their impact should be considered. This point is the superiority of PIF compared to the Attributable Risk measure which presumes deletion of risk factor impact on the outcome condition. This index can be used for ranking risk factors based on the amount of impact that their adjustment has on reducing the incidence of outcomes, which consequentially prioritizes the preventive interventions in health systems and would be more objective and strategic.

In this study we investigated and determined the PIF of each risk factor of diabetes by the amount of feasible reduction. We can infer that modification of combined risk factors of impaired fasting glucose and impaired glucose tolerance is obviously the main priority of preventive interventions designed to reduce the possible incident diabetes. It had the highest PIF among risk factors on the prevention of diabetes (PIF = 0.18).The other most frequent risk factors were impaired fasting glucose, triglycerides, and hypertension with the PIFs of 0.13, 0.13 and 0.12, respectively. The interesting finding based on the results of this study was that the Impact of feasible hypertriglyceridemia modification was more than the impaired glucose tolerance and physical inactivity (PIF = 0.10 for both).

Moreover, the other important finding was that the impact of hypertension (PIF = 0.13) was more than the potential impact of obesity, general obesity, total cholesterol, hypertriglyceridemia and HDL. However, the importance of high blood pressure according to its considerable impact on causing the cardiovascular complications in diabetic patients should be considered.

Intervention to reduce such risk factors in the community demands various effective methods of lifestyle modification. Any intervention should also be evaluated according to internal validity to find out possible biases. More over the external validity is important to be investigated in order to generalize the possible outcomes to different conditions elsewhere.

The improvement of public awareness, encouragements, establishing clear and specific programs, follow up by organizations and advertising are considered to be effective. The improvement of public awareness has different useful ways such as presentations, face to face interviews, publications, the media group, the Internet and panel discussions, which several studies have been discussed the efficacy of these methods previously [25,26] .

But the important point in continuation of this research is the economic issues. Calculation of certain extent in order to optimize the costs for maximum acceptable results is referred as cost-effectiveness. Besides, another issue is regarding the type of diabetes risk factors in patients. In this regard, studies of interventional methods beyond the lifestyle and preventive clinical interventions should be considered. For example, controlled medications for overweight or impaired glucose conditions in terms of cost effectiveness should be further studied and investigated. Another issue is continuing the preventive programs and follow-up of the people who are covered by the program.

The estimated PIF of risk factors is discussed in various diseases especially non-communicable types that preventive methods are typically the best way to fight with them. In a study in South Korea [27] which estimates the potential health outcome by reducing risk factors for stroke, the attributable burden of stroke given the current prevalence of avoidable risk factors and counterfactual prevalence of risk factors are calculated. The burden of stroke in terms of its current prevalence per 100000 people for males were 1940, 4 person-years for smoking, 864.3 person-years for alcohol and 667.3 person-years for high blood pressure. In case of females the current prevalence per 100000 people were 462.8 person – years for alcohol, 455.7 person – years for physical activity and 407.7 person – years for smoking.

In conclusion, adjusted and combined risk factors of impaired fasting glucose and impaired glucose tolerance are the main priority of preventive interventions designed to reduce the possible incidence of diabetes. However, other risk factors mostly hypertension are also not less important and should also be considered in designing interventions. Proper implementation of these interventions will have a significant contribution in reducing the burden of incident diabetes and consequently save money for future health system costs, particularly in the form of national plans to prevent diabetes.

## Endnotes

^a^ The Dietary Approaches to Stop Hypertension

## Appendix

### Formula for calculation of Potential Impact Fraction

(1) $$PIF=\frac{\sum_{i=1}^{n}PiRRi\;-\;\sum_{i=1}^{n}PiRRi}{\sum_{i=1}^{n}PiRRi}$$

P_i_: Primary prevalence

P’_i_: Counterfactual prevalence

RR_i_: Magnitude of effect

## Competing interests

The authors declare that they have no competing interests.

## Authors' contribution

B.L guided the conception of study, funded and supervised the whole work, critically revised the manuscript and was responsible for final approval of the study. R.M was also guided the conception of study and supervised the whole process and also participated in statistical analysis, SM.KM participated in designing the study, gathering and analyzing the data and drafted the manuscript. F.AL participated in systematic literature review and revision of manuscript. F.R participated in systematic literature review and critical revision of study. Z.K participated in provision of study material and writing the manuscript. All authors read and approved the final manuscript.
